# Association Between the Triglyceride–Glucose Index and the Incidence of Diabetes in People With Different Phenotypes of Obesity: A Retrospective Study

**DOI:** 10.3389/fendo.2021.784616

**Published:** 2021-12-09

**Authors:** Su Zou, Chenxi Yang, Rui Shen, Xiang Wei, Junwen Gong, Yali Pan, Yang Lv, Yingjia Xu

**Affiliations:** Department of Cardiology, Shanghai Fifth People’s Hospital, Fudan University, Shanghai, China

**Keywords:** obesity, TyG index, insulin resistance, diabetes, retrospective study

## Abstract

**Aim:**

We aimed to examine the relationship between the Triglyceride–glucose (TyG) index and the incidence of type 2 diabetes in people with different phenotypes of obesity.

**Methods:**

From May 1, 1994 to December 31, 2016, 15,464 participants were enrolled in the medical examination program at the Murakami Memorial Hospital to determine the relationship between the TyG index and the incidence of type 2 diabetes in people with different phenotypes of obesity after 5.38 years of follow-up.

**Results:**

Besides triglycerides, HbA1c%, and FPG, the incidence of type 2 diabetes was found to be significantly associated with the TyG index (p <0.001), age (p <0.001), BMI (p = 0.033), current smoker (p <0.001), and fatty liver (p <0.001). In participants with visceral fat obesity and/or ectopic fat obesity and normal BMI, the TyG index was significantly associated with the incidence of type 2 diabetes after adjusting for confounding factors. In patients with BMI ≥25 mg/m^2^, although there was a trend of the relationship between the TyG index and the incidence of type 2 diabetes, the relationship was no longer positive.

**Conclusion:**

In participants with obesity involving visceral fat obesity and/or fatty liver and normal BMI which is not a measure of body fat distribution, there was a significant association between the TyG index and incidence of T2DM.

## Introduction

Diabetes mellitus type 2 (T2DM) which is an important risk factor of all-cause and cardiovascular death has become a public health problem worldwide (1). The high prevalence of T2DM imposed a heavy socioeconomic burden (2). For early prevention and management, identifying high-risk populations for incident T2DM is needed.

Insulin resistance, a critical pathophysiology pathway of T2DM development, may appear before elevated glucose and impaired glucose regulation (3, 4). The triglyceride–glucose index (TyG index), the product of fasting plasma glucose (FPG) and triglycerides (TGs), has been shown to reflect IR in many studies (5–9). Compared to the Homeostatic Model Assessment of Insulin Resistance (HOMA-IR), the TyG index is more accessible in clinical practice. Moreover, Vasques et al. revealed that the TyG index presented a better performance than the HOMA-IR index in identifying patients with IR (10). Wang et al. found that the TyG index is more strongly associated with arterial stiffness in patients with T2DM in comparison with the HOMA-IR index (11). Many studies identified the association between the TyG index and the risk of new-onset T2DM (8, 12–14).

Obesity which is defined as an excessive amount of body fat has become a global epidemic. T2DM is commonly associated with obesity and obesity is associated with an increased risk of developing T2DM. Most studies that investigated the association between the TyG index and incidence of T2DM enrolled participants regardless of being overweight. A previous study revealed that elevated the TyG index was associated with increased risk of incident T2DM in normal-weight people (14). Body mass index (BMI), an indicator of relative weight for height (weight in kilograms divided by the square of height in meters), is frequently used to assess excess body fat. However, increasing studies showed obesity cannot be evaluated by BMI alone because it cannot present body fat distribution which is more related to IR and clinical outcomes (15–18). To the best of our knowledge, the relationship between TyG index levels and the incidence of T2DM in the different phenotypes of obesity remains unclear. The purpose of this study was to explore the relationship between the TyG index and the incidence of T2DM in people with different phenotypes of obesity.

## Materials and Methods

### Study Design and Study Participants

This was a secondary retrospective study based on NAGALA (NAfld in the Gifu Area, Longitudinal Analysis), and the data was available in the public DRYAD database (www.Datadryad.org) (19). Raw data were provided by Okamura et al. The previous study was approved by the ethics committee of the Murakami Memorial Hospital, and written informed consent for their data to be used was obtained from each participant. Thus, the research ethic was no longer needed due to the public policy statement of the dataset which also obtained the research ethics approval in the previous study. From May 1, 1994 to December 31, 2016, 20,944 participants were enrolled in the medical examination program at the Murakami Memorial Hospital. Participants with: 1) alcoholic fatty liver disease, 2) viral hepatitis (defined by measurements of hepatitis B antigen and hepatitis C antibody), 3) any medication usage at baseline, 4) diabetes at the baseline examination, 5) missing data of covariates, and 6) fasting plasma glucose ≥6.1 mmol/L were excluded. The median follow-up day was 1,967.0 (986.8, 3,425.0).

### Data Collection

A standardized self-administered questionnaire was used to obtain the medical history and lifestyle factors, including physical activity, smoking, and alcohol habits. The standardized spreadsheet was used to collect information on participants. Covariates were assessed at the time of enrolling in this program. Through repeated one to two exams per year, the clinical follow-up was carried out.

### Covariates

The TyG index was calculated as Ln [triglycerides (mg/dl) ∗ fasting glucose (mg/dl)/2] (20). The alcohol consumption was evaluated by asking the participants about the type and amount of alcohol consumption per week during the prior month, then estimating the mean ethanol intake per week. The participants were categorized into the following four groups: no or minimal alcohol consumption (<40 g/week), light (40–140 g/week), moderate (140–280 g/week), or heavy alcohol consumption (>280 g/week). The participants were categorized into three groups by smoking status: never (never smoked cigarettes), past (had smoked in the past but who quit smoking until the baseline visit), or current smoker (smoked at the baseline visit.). The regular exercisers were defined as participants who played any type of sports >1×/week regularly. Fatty liver was diagnosed by the findings of abdominal ultrasonography performed by trained technicians. Obesity was classified according to obesity, visceral fat obesity, and ectopic fat obesity. Obesity was defined as a BMI of ≥25 kg/m^2^ (21–23). Visceral fat obesity was defined as a waist circumference ≥90 cm in men or ≥80 cm in women (24). Ectopic fat obesity was defined as having fatty liver demonstrated by abdominal ultrasound (25, 26). Then, participants was classified into the following eight groups according to the presence (+) or absence (−) of obesity (Ob), visceral fat obesity (Vi), and ectopic fat obesity (Ec): Type 1, Ob−/Vi−/Ec−; Type2, Ob+/Vi−/Ec−; Type 3, Ob−/Vi+/Ec−; Type 4, Ob+/Vi+/Ec−; Type 5, Ob−/Vi−/Ec+; Type 6, Ob+/Vi−/Ec+; Type 7, Ob−/Vi+/Ec+, and Type 8, Ob+/Vi+/Ec+.

### Outcomes

The outcome was the incident T2DM defined as HbA1c ≥6.5%, fasting plasma glucose ≥7 mmol/L, or self-reported.

### Statistical Analysis

Descriptive analysis was applied to all participants. Proportions (%) were used for categorical variables and mean and standard deviation (SD) or median and interquartile range (IQR) was used for continuous variables. The Hazard ratios (HRs) and 95% confidence intervals (CIs) for incidence of T2DM per unit increase were estimated using both univariate and multivariate cox proportional hazards regression models. In addition to the unadjusted model, potential covariates were progressively adjusted in four models. The restricted cubic spline model was used for the dose–response analysis. The cumulative rates of incidence of T2DM were compared using the Kaplan–Meier curves. All statistical analyses were performed using R version 4.0.5 (R Foundation for Statistical Computing, Vienna, Austria). A 2-tailed p-value <0.05) was considered statistically significant.

## Results

**Figure 1:**
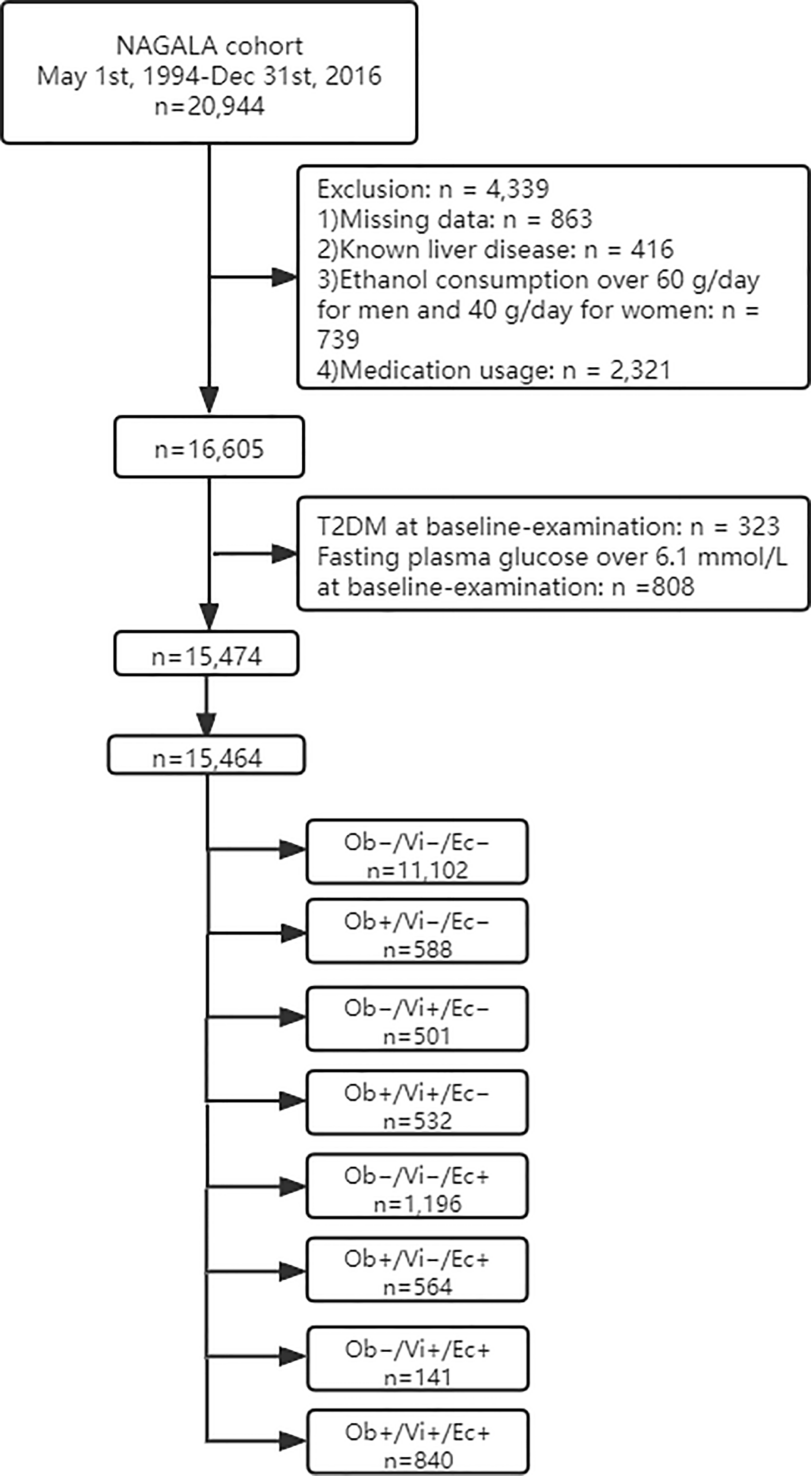
Flowchart of participant selection.

The study flow diagram for the registration of participants is shown in **Figure 1**. Briefly, 20,944 participants were registered in the NAGALA cohort from May 1, 1994 to December 31, 2016 at the Murakami Memorial Hospital (Gifu, Japan). Among them, 863 participants were excluded due to missing data where 416 participants were excluded for known liver disease, 739 participants for heavy drinking habits, and 2,321 participants for baseline medication usage. Also, 323 and 808 participants, who had T2DM and fasting plasma glucose over 6.1 mmol/L at baseline examination respectively, were excluded. A total of 15,464 participants were entered in our study.

Baseline characteristics of participants according to obesity phenotype are summarized in **Table 1**. Overall, 7,034 females (45.5%) and 8,430 males (54.5%) were in this study and there was a significant sex proportion difference in each group. The average age was 43.7 ± 8.9 years old from 43.2 ± 9.0 years old in type 1 to 49.9 ± 7.8 years old in type 7. The baseline TyG index was 8.0 ± 0.6. After an average 5.38 years follow-up, a total of 373 participants (2.4%) developed T2DM. Among them, the proportion in each type was 115/11102 (1%), 14/588 (2.4%), 7/501 (1.4%), 14/532 (2.6%), 61/1196 (5.1%), 46/564 (8.2%), 18/141 (12.8%), and 98/840 (11.7%).

**Table 1:** Baseline characteristics of patients according to obesity phenotype.

	Total	Type 1	Type 2	Type 3	Type 4	Type 5	Type 6	Type 7	Type 8	p value
	(n = 15,464)	(n = 11,102)	(n = 588)	(n = 501)	(n = 532)	(n = 1,196)	(n = 564)	(n = 141)	(n = 840)	
Sex, n (%)										<0.001
female	7,034 (45.5)	5,716 (51.5)	91 (15.5)	453 (90.4)	288 (54.1)	153 (12.8)	24 (4.3)	84 (59.6)	225 (26.8)	
male	8,430 (54.5)	5,386 (48.5)	497 (84.5)	48 (9.6)	244 (45.9)	1043 (87.2)	540 (95.7)	57 (40.4)	615 (73.2)	
Age, years	43.7 ± 8.9	43.2 ± 9.0	43.7 ± 8.5	47.0 ± 9.0	45.2 ± 8.8	45.4 ± 8.3	43.8 ± 8.1	49.9 ± 7.8	43.8 ± 8.2	<0.001
Body Weight, kg	60.6 ± 11.6	56.4 ± 8.9	72.9 ± 7.1	60.4 ± 6.8	74.7 ± 9.7	65.4 ± 7.2	74.6 ± 6.0	65.2 ± 8.4	81.8 ± 11.5	<0.001
Waist circumference, cm	76.5 ± 9.1	72.7 ± 6.8	83.8 ± 4.3	83.4 ± 3.5	90.2 ± 5.6	80.6 ± 4.8	85.5 ± 3.2	86.8 ± 4.9	94.2 ± 6.6	<0.001
BMI, kg/m2	22.1 ± 3.1	20.8 ± 2.1	26.0 ± 0.9	23.1 ± 1.3	27.3 ± 1.9	23.0 ± 1.4	26.3 ± 1.1	23.9 ± 0.9	28.8 ± 2.7	<0.001
Habit of exercise, n (%)	2,709 (17.5)	2,057 (18.5)	116 (19.7)	67 (13.4)	68 (12.8)	182 (15.2)	96 (17)	20 (14.2)	103 (12.3)	<0.001
Smoking status, n (%)										<0.001
Never	9,031 (58.4)	6,843 (61.6)	253 (43)	405 (80.8)	304 (57.1)	505 (42.2)	205 (36.3)	93 (66)	423 (50.4)	
Past	2,952 (19.1)	1,927 (17.4)	146 (24.8)	50 (10)	103 (19.4)	347 (29)	180 (31.9)	25 (17.7)	174 (20.7)	
Current	3,481 (22.5)	2,332 (21)	189 (32.1)	46 (9.2)	125 (23.5)	344 (28.8)	179 (31.7)	23 (16.3)	243 (28.9)	
Ethanol consumption, g/wk	47.8 ± 82.3	46.0 ± 80.0	73.9 ± 99.7	27.5 ± 59.3	58.0 ± 96.5	52.4 ± 87.9	56.3 ± 89.3	41.5 ± 74.9	46.8 ± 84.1	<0.001
Alcohol consumption, n (%)										<0.001
Non	11,805 (76.3)	8,518 (76.7)	384 (65.3)	427 (85.2)	388 (72.9)	901 (75.3)	422 (74.8)	109 (77.3)	656 (78.1)	
Light	1,758 (11.4)	1,279 (11.5)	84 (14.3)	47 (9.4)	62 (11.7)	125 (10.5)	59 (10.5)	16 (11.3)	86 (10.2)	
Moderate	1,360 (8.8)	955 (8.6)	81 (13.8)	22 (4.4)	52 (9.8)	113 (9.4)	57 (10.1)	14 (9.9)	66 (7.9)	
Heavy	541 (3.5)	350 (3.2)	39 (6.6)	5 (1)	30 (5.6)	57 (4.8)	26 (4.6)	2 (1.4)	32 (3.8)	
FPG, mg/dl	93.0 ± 7.4	91.7 ± 7.3	96.1 ± 6.5	92.2 ± 6.9	95.3 ± 6.9	96.8 ± 6.8	97.8 ± 6.2	96.6 ± 6.4	97.8 ± 6.5	<0.001
HbA1c, %	5.2 ± 0.3	5.1 ± 0.3	5.2 ± 0.3	5.2 ± 0.3	5.3 ± 0.3	5.3 ± 0.3	5.3 ± 0.3	5.4 ± 0.3	5.3 ± 0.3	<0.001
HDL cholesterol, mg/dL	56.5 ± 15.6	59.7 ± 15.5	48.7 ± 11.3	58.6 ± 14.2	51.1 ± 12.9	47.5 ± 12.3	43.5 ± 9.7	51.0 ± 12.6	44.8 ± 9.9	<0.001
Total Cholesterol, mg/dL	196.0 (174.0, 219.0)	192.0 (171.0, 215.0)	198.0 (177.0, 223.0)	203.0 (180.0, 226.0)	205.0 (185.0, 227.2)	208.0 (186.0, 230.0)	209.0 (186.0, 231.0)	214.0 (189.0, 234.0)	213.0 (190.0, 234.0)	<0.001
Triglycerides, mg/dL	80.8 ± 58.1	67.7 ± 46.1	99.5 ± 57.2	70.0 ± 40.2	96.4 ± 58.6	122.3 ± 73.0	138.3 ± 77.3	103.0 ± 53.1	136.2 ± 76.6	<0.001
ALT, IU/L	17.0 (13.0, 23.0)	15.0 (12.0, 20.0)	21.0 (16.0, 28.0)	15.0 (12.0, 18.0)	18.0 (14.0, 25.0)	25.0 (19.0, 34.0)	31.0 (23.0, 41.0)	22.0 (16.0, 29.0)	30.0 (22.0, 46.0)	<0.001
AST, IU/L	17.0 (14.0, 21.0)	17.0 (14.0, 20.0)	18.0 (14.0, 22.0)	17.0 (14.0, 20.0)	18.0 (14.0, 21.0)	19.0 (16.0, 24.0)	22.0 (17.0, 27.0)	19.0 (16.0, 22.0)	22.0 (18.0, 28.0)	<0.001
GGT, IU/L	15.0 (11.0, 22.0)	14.0 (11.0, 19.0)	20.0 (14.0, 29.0)	13.0 (11.0, 17.0)	17.0 (12.0, 27.0)	22.0 (16.0, 32.0)	26.0 (19.0, 38.2)	19.0 (15.0, 27.0)	25.0 (17.0, 37.0)	<0.001
SBP, mmHg	114.5 ± 15.0	111.3 ± 13.8	122.7 ± 13.8	115.6 ± 14.6	123.4 ± 15.0	119.8 ± 14.0	125.3 ± 13.1	120.8 ± 13.8	129.0 ± 15.4	<0.001
DBP, mmHg	71.6 ± 10.5	69.4 ± 9.7	77.3 ± 9.6	71.4 ± 10.0	77.3 ± 10.3	75.6 ± 9.8	79.4 ± 9.2	76.0 ± 9.2	81.3 ± 10.5	<0.001
TyG	8.0 ± 0.6	7.9 ± 0.6	8.3 ± 0.5	7.9 ± 0.5	8.3 ± 0.6	8.5 ± 0.6	8.7 ± 0.5	8.4 ± 0.5	8.7 ± 0.5	<0.001
Follow up duration, days	1,967.0 (986.8, 3,425.0)	2,041.0 (1,020.0, 3,492.0)	1,943.0 (979.8, 3,608.0)	1,779.0 (1,058.0, 2,881.0)	1,820.0 (770.0, 3,232.0)	2114.0 (997.5, 3,644.0)	2,061.0 (918.0, 3,376.0)	2060.0 (847.0, 3,333.0)	1,823.0 (752.8, 3,282.0)	<0.001
Incident DM, n (%)	373 (2.4)	115 (1)	14 (2.4)	7 (1.4)	14 (2.6)	61 (5.1)	46 (8.2)	18 (12.8)	98 (11.7)	<0.001

Data were mean ± SD or median (IQR) for skewed variables or numbers (proportions) for categorical variables.

ALT, alanine aminotransferase; AST, aspartate aminotransferase; BMI, Body Mass Index; DBP, diastolic blood pressure; DM, diabetes mellitus; FPG, fasting plasma glucose; GGT, gamma-glutamyl transferase; HbA1c, glycated hemoglobin; HDL, high density lipoproteins; SBP, systolic blood pressure; TyG, Triglyceride–glucose.

**Table 2** shows the univariate and multivariate analyses and predictors of incidence of T2DM. The univariate analysis showed that almost all variables in this table were associated with the incidence of T2DM except with the habit of exercise, light, and moderate alcohol consumption, and type 3 obesity (only BMI ≥25 kg/m^2^). However, after adjusting for all variables except triglycerides, HbA1c%, and FPG which are related to the TyG index, only TyG (HR 1.70, 95%CI 1.34–2.16, p <0.001), age (HR 1.06, 95%CI 1.04–1.07, p <0.001), BMI (HR 1.07, 95%CI 1.01–1.14, p = 0.033), current smoker (HR 1.76, 95%CI 1.34–2.30, p <0.001), and fatty liver (HR 2.76, 95%CI 2.13–3.58, p <0.001) were associated with the incidence of T2DM.

**Table 2:** Result of univariate and multivariate analysis and predictors of incidence of DM.

	Univariate analysis		Multivariate analysis	
	HR (95%CI)	P value	HR (95%CI)	P value
TyG	3.76 (3.22, 4.38)	<0.001	1.70 (1.34–2.16)	<0.001
Sex	2.52 (1.98, 3.21)	<0.001	0.63 (0.45–0.89)	0.008
Age, years	1.06 (1.04, 1.07)	<0.001	1.06 (1.04–1.07)	<0.001
Body Weight, kg	1.06 (1.05, 1.06)	<0.001		
Waist circumference, cm	1.09 (1.08, 1.1)	<0.001	1.02 (1.00–1.05)	0.072
BMI, kg/m^2^	1.24 (1.22, 1.27)	<0.001	1.07 (1.01–1.14)	0.033
Habit of exercise	0.76 (0.56, 1.02)	0.064	0.89 (0.66–1.20)	0.455
Smoking status				
Never	Ref			
Past	1.66 (1.26, 2.18)	<0.001	0.94 (0.68–1.28)	0.690
Current	2.58 (2.06, 3.24)	<0.001	1.76 (1.34–2.30)	<0.001
Ethanol consumption, g/wk	1.0017 (1.0007, 1.0028)	0.001	1.00 (1.00–1.00)	0.682
Alcohol consumption				
Non	Ref			
Light	0.9 (0.65, 1.26)	0.551		
Moderate	1.15 (0.82, 1.62)	0.424		
Heavy	2.24 (1.54, 3.27)	<0.001		
FPG, mg/dl	1.2 (1.18, 1.22)	<0.001		
HbA1c%	54.3 (39.51, 74.62)	<0.001		
HDL cholesterol, mg/dl	0.95 (0.94, 0.96)	<0.001	0.99 (0.98–1.00)	0.214
Total Cholesterol, mg/dl	1.01 (1.01, 1.01)	<0.001	1.00 (1.00–1.00)	0.912
Triglycerides, mg/dl	1.0066 (1.0059, 1.0074)	<0.001		
ALT, IU/L	1.0063 (1.0052, 1.0074)	<0.001	1.01 (1.00–1.02)	0.165
AST, IU/L	1.0083 (1.0062, 1.0104)	<0.001	1.00 (0.98–1.01)	0.616
GGT, IU/L	1.01 (1.01, 1.01)	<0.001	1.00 (1.00–1.01)	0.158
SBP, mmHg	1.03 (1.03, 1.04)	<0.001	1.00 (0.98–1.02)	0.974
DBP, mmHg	1.05 (1.04, 1.06)	<0.001	1.00 (0.98–1.03)	0.938
Fatty.liver.0.1: 1 *vs* 0	7.02 (5.7, 8.63)	<0.001	2.76 (2.13–3.58)	<0.001
Type				
1	ref			
2	2.26 (1.3, 3.94)	0.004		
3	1.74 (0.81, 3.73)	0.156		
4	2.8 (1.6, 4.87)	<0.001		
5	4.78 (3.51, 6.52)	<0.001		
6	8.05 (5.72, 11.33)	<0.001		
7	13.13 (7.98, 21.58)	<0.001		
8	12.65 (9.66, 16.57)	<0.001		

ALT, alanine aminotransferase; AST, aspartate aminotransferase; BMI, Body Mass Index; DBP, diastolic blood pressure; DM, diabetes mellitus; FPG, fasting plasma glucose; GGT, gamma-glutamyl transferase; HbA1c, glycated hemoglobin; HDL, high density lipoproteins; SBP, systolic blood pressure; TyG, Triglyceride–glucose.

**Figure 2:**
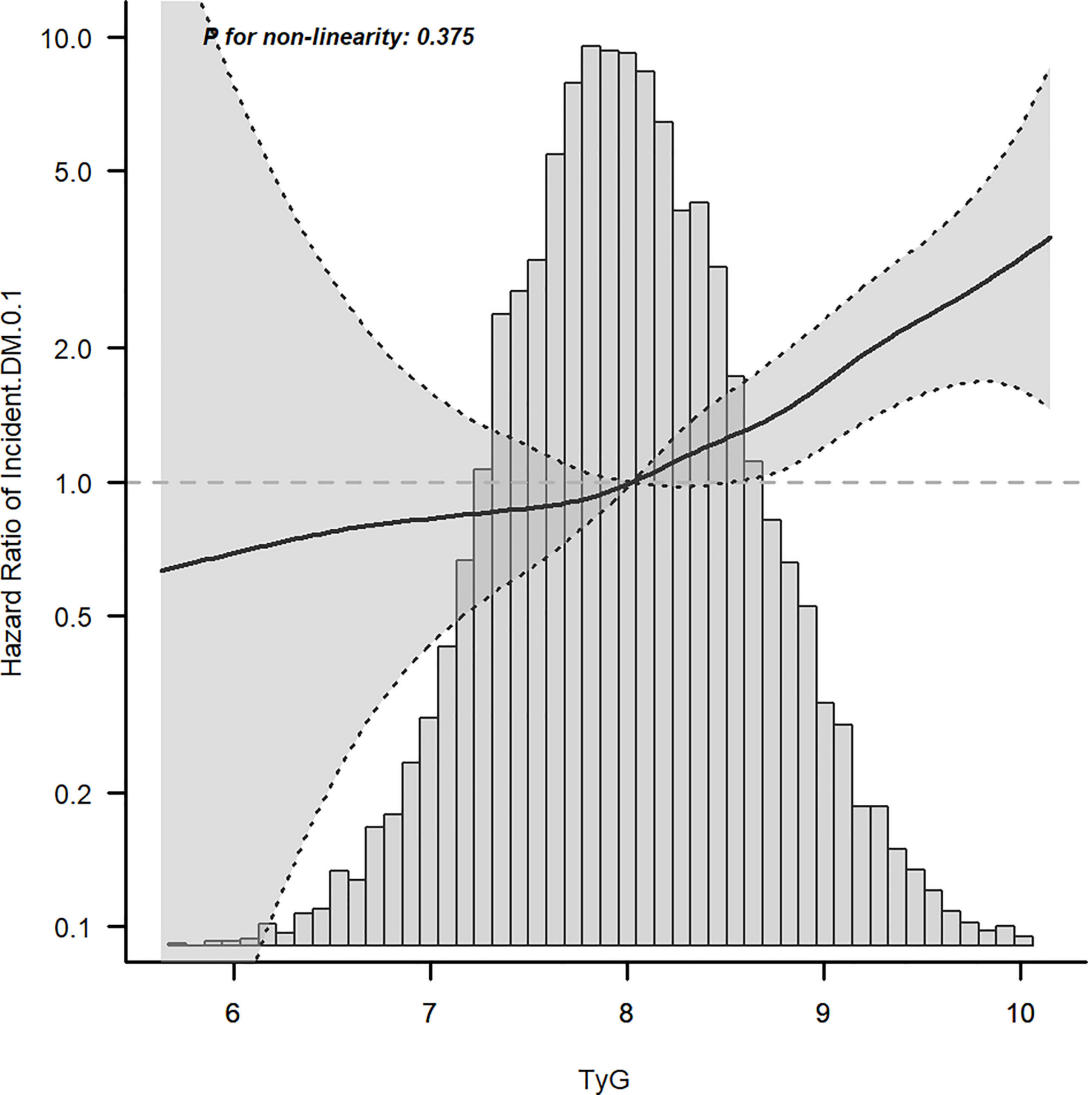
Dose–response relationship between the TyG index and incident T2DM after adjusting for model 3.

**Figure 3:**
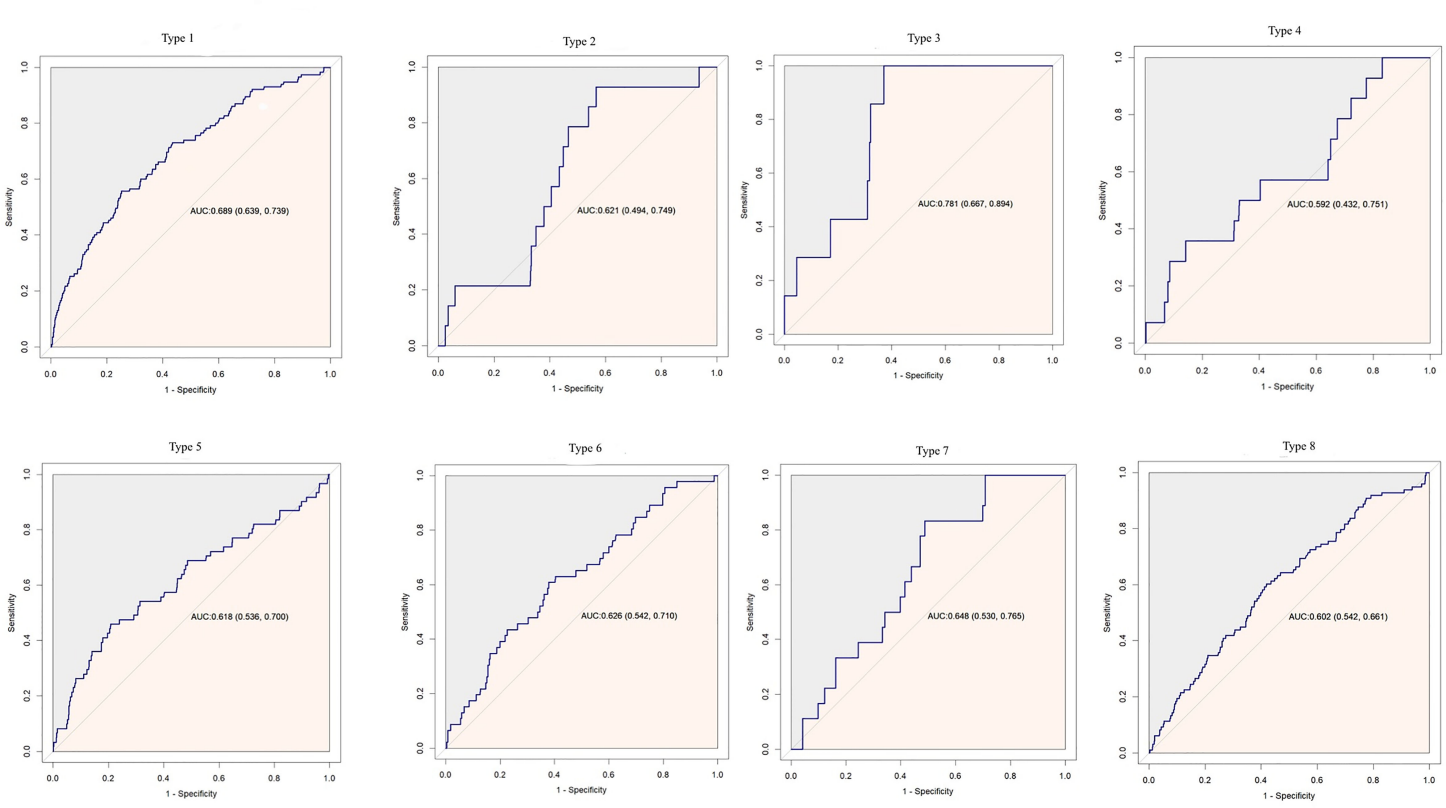
Receiver operating characteristic curve analysis of the TyG index to predict incident T2DM in different types of obesity. AUC, area under curve.

Cox proportional hazard models with robust estimator were fit to examine the association between the TyG index and the incidence of T2DM in three models which included different potential covariates of sex, age, ethanol consumption, fatty liver, BMI, waist circumference, the habit of exercise, gamma-glutamyl transferase (GGT), alanine aminotransferase (ALT), aspartate aminotransferase (AST), high-density lipoprotein cholesterol (HDL-C), total cholesterol (TC), smoking status, systolic blood pressure (SBP), and diastolic blood pressure (DBP). We found that the TyG index was independent associated with the incidence of T2DM in all three models (all p <0.001) (**Table 3**). The dose–response analysis with a restricted cubic spline model showed a nearly linear relationship between the TyG index and the incidence of T2DM after adjustment for potential covariates in Model 3 (**Figure 2**). We found an increasing trend of incidence of T2DM with a higher TyG index despite the lack of a linear relationship between the TyG index and the incidence of T2DM. The ROC curves of the TyG index as a marker to predict the incidence of T2DM in different types of obesity are illustrated in **Figure 3**. The AUC of the TyG index for predicting the occurrence of T2DM was 0.750 (95% CI 0.726–0.775) in overall participants and the AUC of TyG index for predicting the incidence of T2DM was 0.689 (95%CI 0.639–0.739), 0.621 (95%CI 0.494–0.749), 0.781 (95%CI 0.667–0.894), 0.592 (95%CI 0.432–0.751), 0.618 (95%CI 0.536–0.700), 0.626 (95%CI 0.542–0.710), 0.648 (95%CI 0.530–0.765), and 0.602 (95%CI 0.542–0.661) according to different types of obesity.

**Table 3:** Multivariable-adjust HRs and 95%CI of the TyG index on incidence of diabetes mellitus.

TyG index		Unadjusted	Model 1	Model 2	Model 3
		HR (95%CI)	HR (95%CI)	HR (95%CI)	HR (95%CI)
overall TyG	continuous per unit increase	3.76 (3.22–4.38)	3.38 (2.85–4.01)	1.89 (1.56–2.30)	1.70 (1.34–2.16)
	p value	<0.001	<0.001	<0.001	<0.001

Data presented were HRs and 95%CIs.

Model 1 adjust for sex and age.

Model 2 adjust for sex, age ethanol consumption, fatty liver, body mass index and smoking status.

Model 3 adjust for sex, age, ethanol consumption, fatty liver, body mass index, waist circumference, habit of exercise, gamma-glutamyl transferase, alanine aminotransferase, aspartate aminotransferase, high density lipoproteins cholesterol, total cholesterol, smoking status, systolic blood pressure and diastolic blood pressure.

**Figure 4:**
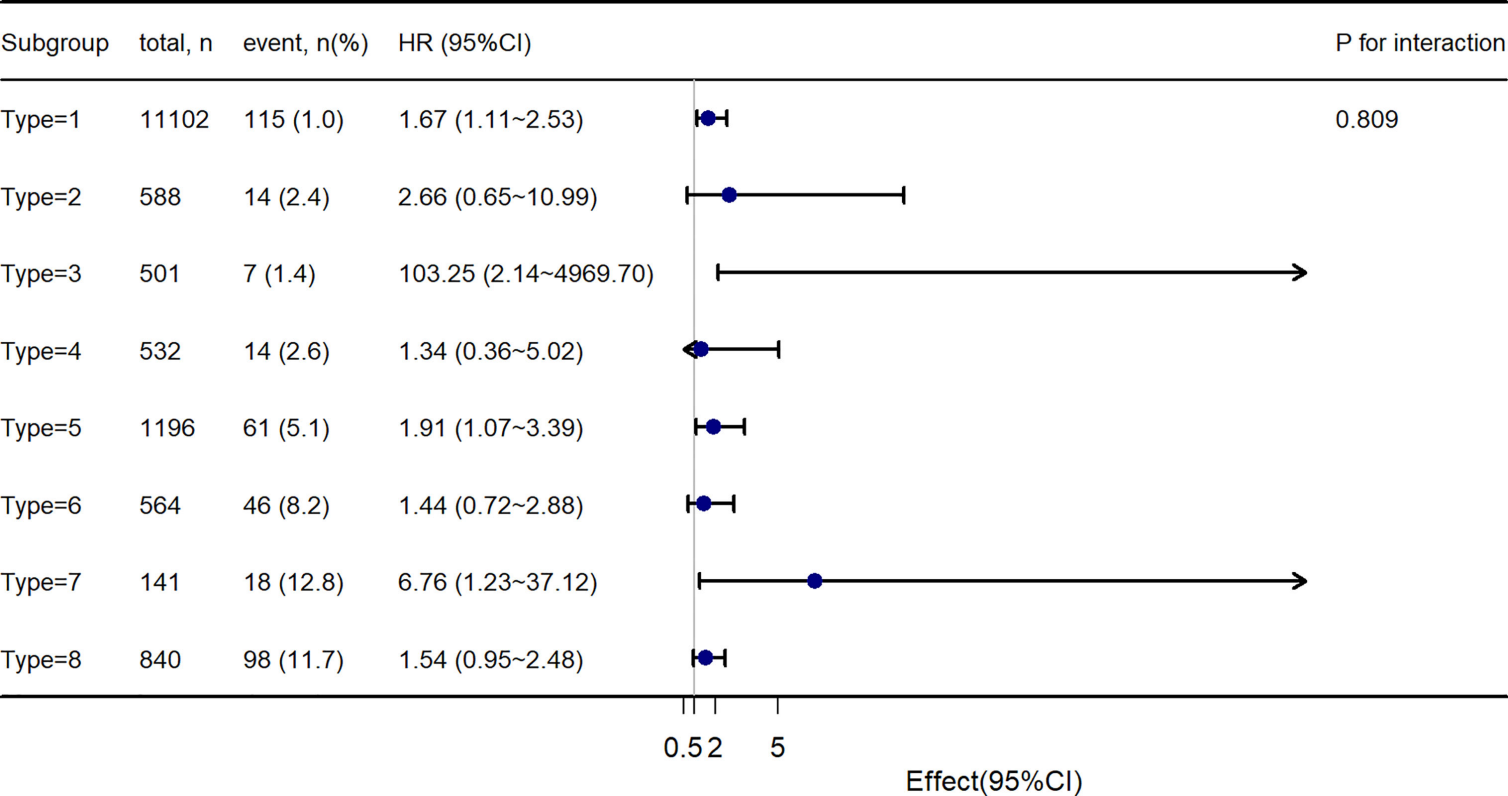
Stratification analysis on the association of TyG index with incident T2DM.

**Figure 5:**
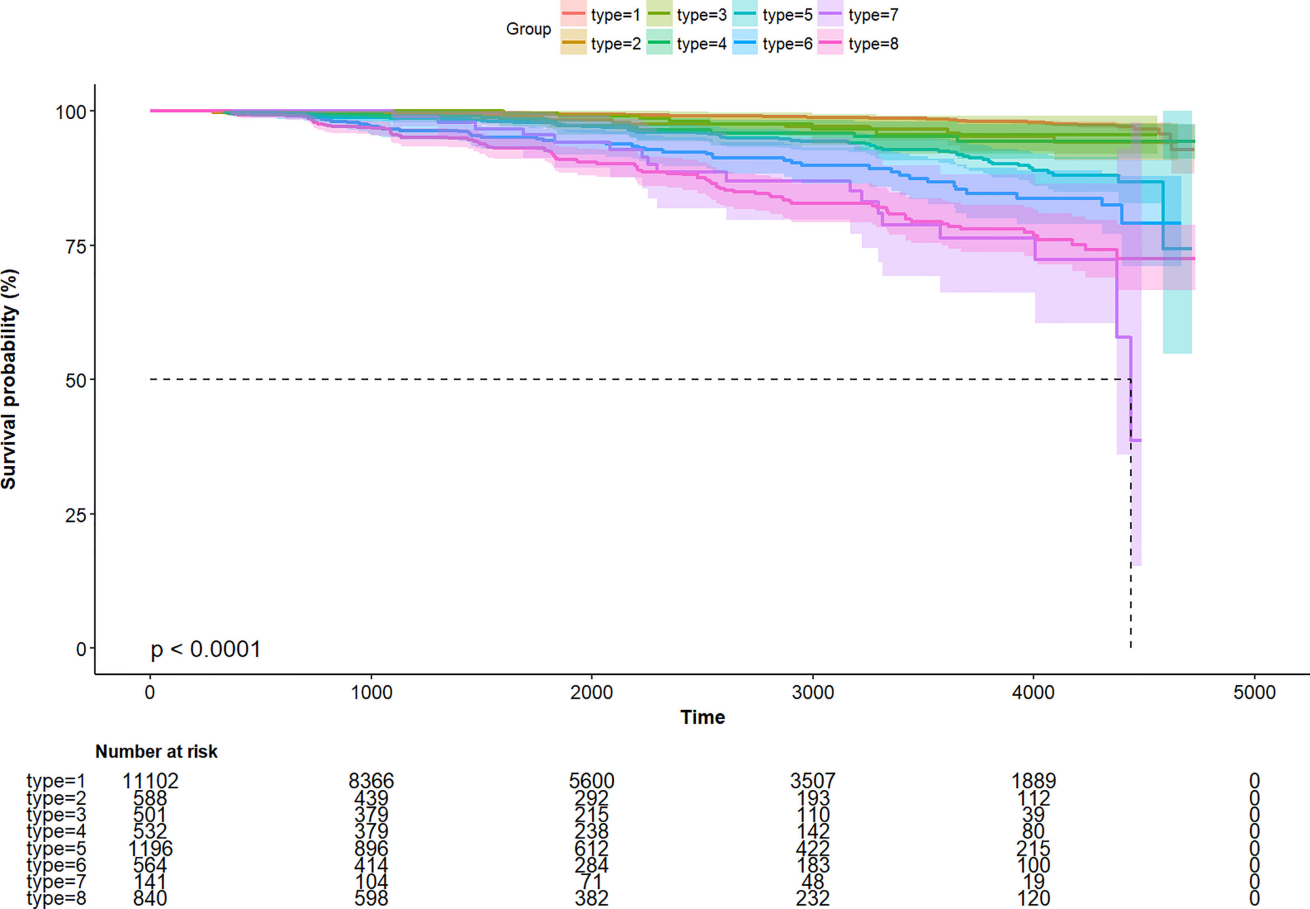
Kaplan–Meier curve of time to incident diabetes during follow-up.

Further, to assess the association of the TyG index with the incidence of T2DM in different obesity phenotypes, we performed Cox proportional-hazards regression models (**Figure 4**). After adjusting for model 3, we found that there were different HR and 95%CI. In participants with Type 1, Type3, Type 5, and Type 7 obesity, the TyG index was significant associated with the incidence of T2DM. In Type 2, Type 4, Type 6, and Type 8 obesity, although there was a trend of the relationship between the TyG index and the incidence of T2DM, the relationship was no longer positive. And the multiplicative interactions of the TyG index × obesity phenotypes (p for interaction = 0.809) regarding the risk of incident T2DM were not significant. Then, the cumulative rates of incidence of T2DM were compared using the Kaplan–Meier curves (**Figure 5**). Participants with Type 7 and Type 8 obesity had a higher incidence of T2DM than others.

## Discussion

Using a dataset from NAGALA, we identified that the TyG index was independently associated with the incidence of T2DM after an average of 5.38 years follow-up. In subgroup analysis according to different phenotypes of obesity or non-obesity, we found that different phenotypes of obesity had different hazard ratios on the TyG index for predicting the occurrence of T2DM. In people with visceral fat obesity and/or ectopic fat obesity, and BMI less than 25 kg/m^2^, the TyG index was independently associated with the incidence of T2DM. However, in people with excess BMI, the relationship was no longer positive.

The TyG index, the product of triacylglycerol and glucose, has been used as an early marker of insulin resistance and an inexpensive and reliable indicator to identify T2DM (7, 10, 27, 28). According to cox proportional hazards regression models, the TyG index was significantly associated with the incidence of T2DM, well in line with previous studies. Zhang et al. found that increasing the TyG index elevated the risk of incident T2DM after six years of follow-up among 5,706 Chinese participants (14). Lee et al. revealed that a higher TyG index had a higher risk for developing T2DM in a Korean prospective cohort study during a median of 4.6 years of follow-up (29). Furthermore, Navarro-Gonzalez et al. found that the TyG index was useful for early identification of patients at risk of T2DM during 10 years follow-up in a large white European population (28).

Obesity, defined as an excessive amount of body fat, is considered a major risk factor for T2DM. In clinical practice, obese individuals refer to people who have elevated BMI, which is expressed as the ratio of body weight (kilograms) by height (meter) in the square. However, increasing studies showed obesity cannot be evaluated by BMI alone because it represents a heterogeneous entity. Okamura et al. found that ectopic fat obesity presented a greater risk for incident T2DM than BMI. Moreover, it is famous that there is an obesity paradox in cardiovascular diseases, namely, obesity may be associated with lower all-cause and cardiovascular mortality (30). One manifestation of the obesity paradox was increased BMI patients had lower mortality compared with normal-weight participants and there was an association between weight loss and increased morbidity and mortality among participants with T2DM and cardiovascular mortality (31). Although an elevated BMI increases the risk of health complications, not every overweight or obese people develop risk factors or health issue. “Metabolically healthy” or “fit fat” obesity may exist under the definition of obesity from BMI. The regional distribution of body fat may much more important than adipose tissue mass.

In our investigation, we confirmed that the TyG index was independently associated with the incidence of T2DM with a 5.38-year follow-up. Then, to exam the association between the TyG index and the incidence of T2DM in different phenotypes of obesity or non-obesity, we divided obesity into seven groups according to BMI, waist circumference, and fatty liver demonstrated by abdominal ultrasound. There were eight groups in total. We found that there were different HR and 95%CI. In participants with Type 1, Type3, Type 5, and Type 7 obesity, the TyG index was significant associated with the incidence of T2DM. In Type 2, Type 4, Type 6, and Type 8 obesity, although there was a trend of the relationship between the TyG index and the incidence of T2DM, the relationship was no longer positive. And the AUC of TyG index for predicting the incidence of T2DM was different regarding different phenotypes of obesity. Briefly, in people only with visceral fat obesity and/or ectopic fat obesity, and BMI less than 25 kg/m^2^, the TyG index could predict the incidence of T2DM. However, in people with excess BMI, the prediction value was no longer positive. Our finding of significant association between the TyG index and the incidence of T2DM in non-obesity people was in line with the previous study (32).

The mechanism underlying the association between the TyG index and the incidence of T2DM has been explained by insulin resistance in many studies. The TyG index is a strong indicator of insulin resistance. Moreover, the different adipogenic capacities of preadipocytes may explain the different HR and 95%CI of different phenotypes of obesity or non-obesity. Preadipocytes from the visceral fat depots had lower adipogenic capacity compared with preadipocytes from the subcutaneous fat depots (33). Furthermore, low adipogenic capacity had been related to metabolic abnormalities featured with insulin resistance and poor lipid profile (34). Because the liver plays a critical role in glucose and lipid metabolism, the fatty liver may be a key driver of the dysmetabolic state. So, excess visceral adiposity and ectopic fat accumulation are the main cause of insulin resistance. In our study, in participants with obesity involving visceral fat obesity and fatty liver, but not excess BMI which is not a measure of body fat distribution, there was a significant association between the TyG index and incidence of T2DM. BMI may not a best obesity marker for predicting disease. Though the participants with Type 2, Type 4, and Type 6 obesity have a higher proportion of T2DM, those types involving BMI may reduce the risk to some extent.

There are some limitations to our study. First, the current study only included the Japanese population, and the interpretation of the finding to the non-Japanese population may be limited. Second, as an observational study, the present findings of the relationship between the TyG index and the risk of incidence of T2DM may preclude conclusions about causality. Third, due to the database limitations, lipid, glucose, and other parameters were only measured once and the follow-up time was no longer enough. So, the potential errors may bias the findings. Fourth, though the long follow-up time was carried out in participants, the mean age was 43.7 ± 8.9 years old. So, the incidence of type 2 diabetes was relatively low. This may bias the finding. Fifth, in medical examination, OGTT is not a routine item. Some participants with high postprandial plasma glucose would be missed at baseline and follow-up.

Despite the aforementioned limitations, this study had several strengths. First, to the best of our knowledge, this is the first study to investigate the association between the TyG index and the risk of incidence of T2DM in participants with different phenotypes of obesity. Second, the dataset extracted from the NAGALA database was relatively large and complete. Third, our analysis of the relationship between the TyG index and incident T2DM in participants with different phenotypes of obesity was adjusted for more potential confounding factors than previous studies. We did try our best to overcome potential shortcomings to make the result more robust and reliable.

## Conclusion

In participants with obesity involving visceral fat obesity and fatty liver which is a measure of body fat distribution, but not excess BMI, there was a significant association between the TyG index and the incidence of T2DM.

## Data Availability Statement

The datasets presented in this study can be found in online repositories. The names of the repository/repositories and accession number(s) can be found below: “Okamura, Takuro et al. (2019), Data from: Ectopic fat obesity presents the greatest risk for incident type 2 diabetes: A population-based longitudinal study, Dryad, Dataset, https://doi.org/10.5061/dryad.8q0p192”.

## Ethics Statement

The studies involving human participants were reviewed and approved by the Ethics Committee of the Murakami Memorial Hospital. The ethics committee waived the requirement of written informed consent for participation.

## Author Contributions

SZ and YX designed the study. SZ, CY, and RS collected the data. SZ, CY, XW, and JG analyzed the data. SZ, CY, RS, XW, JG, YP, and YL interpreted the result. SZ wrote the first draft of the manuscript. YX contributed to the refinement of the manuscript. All authors contributed to the article and approved the submitted version.

## Funding

This work was supported by the Shanghai Minhang Science and Technology Commission [grant number 2020WYZD07] and the Science and Technology Commission of Shanghai Municipality [grant numbers 19401970200 and 19411971900].

## Conflict of Interest

The authors declare that the research was conducted in the absence of any commercial or financial relationships that could be construed as a potential conflict of interest.

## Publisher’s Note

All claims expressed in this article are solely those of the authors and do not necessarily represent those of their affiliated organizations, or those of the publisher, the editors and the reviewers. Any product that may be evaluated in this article, or claim that may be made by its manufacturer, is not guaranteed or endorsed by the publisher.
